# An Automatic Classification System for Environmental Sound in Smart Cities

**DOI:** 10.3390/s23156823

**Published:** 2023-07-31

**Authors:** Dongping Zhang, Ziyin Zhong, Yuejian Xia, Zhutao Wang, Wenbo Xiong

**Affiliations:** 1 Key Laboratory of Electromagnetic Wave Information Technology and Metrology of Zhejiang Province, China Jiliang University, Hangzhou 310018, China; s21030810012@cjlu.edu.cn (Z.Z.); s21030810009@cjlu.edu.cn (Y.X.); p21030854056@cjlu.edu.cn (Z.W.); 2 Hangzhou Aihua Intelligent Technology Co., Ltd., 359 Shuxin Road, Hangzhou 311100, China; xiongwenbo@aihua-ai.onaliyun.com

**Keywords:** environment sound classification, convolutional neural networks, data processing, residual network

## Abstract

With the continuous promotion of “smart cities” worldwide, the approach to be used in combining smart cities with modern advanced technologies (Internet of Things, cloud computing, artificial intelligence) has become a hot topic. However, due to the non-stationary nature of environmental sound and the interference of urban noise, it is challenging to fully extract features from the model with a single input and achieve ideal classification results, even with deep learning methods. To improve the recognition accuracy of ESC (environmental sound classification), we propose a dual-branch residual network (dual-resnet) based on feature fusion. Furthermore, in terms of data pre-processing, a loop-padding method is proposed to patch shorter data, enabling it to obtain more useful information. At the same time, in order to prevent the occurrence of overfitting, we use the time-frequency data enhancement method to expand the dataset. After uniform pre-processing of all the original audio, the dual-branch residual network automatically extracts the frequency domain features of the log-Mel spectrogram and log-spectrogram. Then, the two different audio features are fused to make the representation of the audio features more comprehensive. The experimental results show that compared with other models, the classification accuracy of the UrbanSound8k dataset has been improved to different degrees.

## 1. Introduction

Convolutional neural networks can be traced back to the 1980s [1], but they were not used for classification tasks until 2010, gradually replacing traditional manual features. Convolutional neural networks have made significant progress in many pattern recognition tasks, including image classification, re-identification [2,3] and fault detection and diagnosis (FDD) [4]. With the continuous development of deep learning technology, the use of neural network technology for image and signal processing has become the choice of more and more researchers [5,6]. Especially in speech- and audio-related tasks [7,8], neural network techniques have performed better than traditional machine learning algorithms. Neural networks extract critical features from audio signals to classify ambient sounds efficiently and accurately [9,10,11]. In recent years, many scholars have combined attention mechanisms with neural networks to emphasize certain partial features to further improve performance [12,13]. Some conventional machine learning techniques, such as support vector machines (SVM) [14], random forest [15], K Nearest Neighbors (KNN) [16], and other classifiers, have achieved specific results in recent research [17,18]. These traditional classification algorithms still face challenges in acquiring ideal identifying and classifying results in the face of complex and changeable environmental sounds. Therefore, building a more robust and efficient neural network has become a new target for sound recognition and classification.

In the face of one-dimensional dynamic sound signals, how to perform better recognition and classification also comes down to how to choose a better feature representation. The currently commonly used feature representations can be divided into features directly extracted from the original signal in the time domain (such as autocorrelation, zero-crossing rate) [19,20] and features obtained by converting the signal into frequency (such as spectral centroid [21], Mel-scale frequency cepstral coefficient). The most widely used feature extraction technique is the Mel-scale frequency cepstral coefficient (MFCC) [22], but it also shows some limitations when dealing with low signal-to-noise ratio (SNR) sound signals. Therefore, Jitong Chen et al. introduced gammatone frequency cepstral coefficients (GFCC) feature extraction technology on this basis to better simulate the frequency division characteristics of the human ear to suppress background noise better [23]. However, achieving the ideal classification effect using a single traditional feature in the face of complex and changeable natural environmental sounds is challenging in practical applications. Therefore, Ruixue Li et al. proposed an environmental sound classification method based on multi-feature fusion, which captures audio features from two different aspects of signal time and frequency domains; feature fusion is conducted between GFCC characteristics based on human ear auditory characteristics and short-time energy characteristics [24]. This shows that fused features are more suitable for classification than single features. Hence, it is necessary to propose an effective feature fusion method to analyze complex ambient acoustic signals.

As mentioned, the current simple neural network cannot perform high-efficiency classification, and a single feature cannot complete the high-precision sort. In this project, we propose a dual-branch residual network system based on feature fusion to classify urban ambient sounds automatically. The proposed method unifies and pre-processes the audio signal, then extracts spectral features and log-Mel features separately and passes these two features to a dual-branch residual network. Finally, feature fusion is performed using adaptive weights and passed to the classification layer for final classification. We show that the representation generated by the proposed method has a specific improvement in average accuracy compared with other models.

The rest of the paper is organized as follows. Section 2 introduces the related work, and Section 3 explains the method overview and implementation. Section 4 shows the experimental evaluation of our method. Section 5 concludes the paper.

## 2. Related Work

Nowadays, extensive research on deep learning has greatly promoted the development of artificial intelligence and machine learning. Deep learning has great potential in many fields, such as natural language processing and computer vision. Therefore, more and more researchers are beginning to introduce this technology into ESC research. For example, in 2015, Piczak et al. [9] proposed a sound classification network model consisting of two convolutional layers with maximum pooling and two fully connected layers, and utilized the frequency domain characteristics of the common dataset for audio recognition, thereby verifying the feasibility of convolutional neural networks in ESC problems. In subsequent work, Tokozume et al. [11] proposed a novel end-to-end ESC system using convolutional neural networks (CNN), which improved classification accuracy by 6.5% compared to the log-Mel CNN proposed by Piczak et al. In 2019, Chen et al. introduced dilated CNN into the ESC problem and achieved better results compared to using maximum pooling CNN. They also explored the effects of different expansion rates and expansion convolution layers on experimental results. In 2021, Mu et al. [12] proposed a convolutional neural network model based on time-frequency attention, which can more effectively learn time and frequency features from log-Mel spectrograms. Recently, Wang et al. [13] proposed an effective and lighter convolutional neural network structure with multi-channel temporal attention blocks; this structure applies temporal attention mechanisms to extract time information related to each channel, in order to utilize channel time information more fully. The work inspiration proposed in this article comes from Li et al.’s work [24], which explores the potential of feature fusion. Compared to a single feature, multiple features have better frequency domain information representation.

## 3. Proposed Method

**Figure 1:**
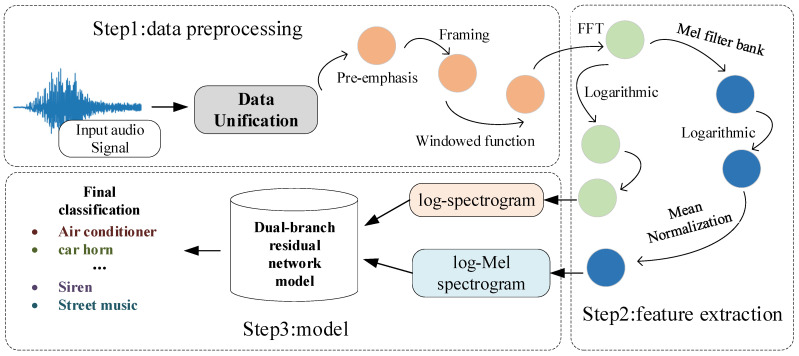
Model Architecture.

In the proposed method, the sound signal is first passed through the data pre-processing block. The processed data are then subjected to feature capture to obtain log-spectrogram and log-Mel spectrogram features. Then, these two features are transmitted to the dual-branch residual network for feature extraction and fusion, and finally, pass to the classification layer to output the final classification results. The proposed system is presented as shown in Figure 1.

### 3.1. Data Pre-Processing

Before analyzing audio signals, pre-processing operations such as pre-emphasis, framing, and windowing must be performed. The purpose of these operations is to eliminate the impact of external noise and factors such as aliasing, high-order harmonic distortion, and high-frequency caused by the equipment used to collect voice signals on the quality of audio signals. This is to try to ensure that the subsequent audio processing results in a more uniform and smoother signal, providing high-quality parameters for signal parameter extraction and improving the quality of audio processing.

**Figure 2:**
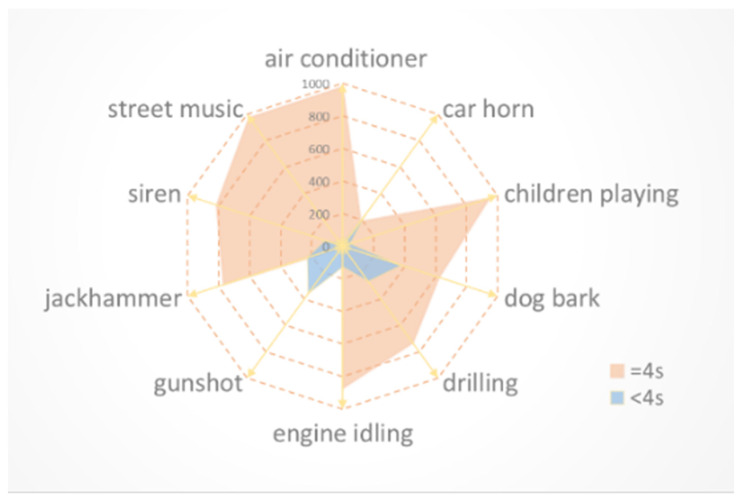
All samples of the UrbanSound8K dataset.

Since the public dataset UrbanSound8K [25] used in this paper contains data greater than 4 s, and less than 1 s, audio signals that are too long or short are not conducive to experiments. As shown in Figure 2, there are several categories (such as gunshot and dog bark) with a sample size of less than 4 s that account for an excessive proportion. Directly discarding these sample data during the pre-processing stage would be a huge waste. Some methods, such as zero padding and random padding, have been used to patch the data. Still, zero padding simply fills the data matrix to a particular length using zero values. Therefore, a simple and effective data-patching strategy is proposed, called the loop-padding method, described below. If the number of sampling points of input data is greater than the specified number of sampling points, [0, the specified number of sampling points] is returned. If the number of sampling points in the input data is less than the specified number of sampling points, a 0-filled array Y_new_ of the given length and type is generated to perform data filling. Starting from one bit after the number of sampling points in the input data, until the specified number of sampling points is reached, all the data filled in the loop are inserted into the array Y_new_. Finally, the output array Y_new_ is the data filled in the loop. Its pseudo code is shown in Algorithm 1. The data are then uniformly pre-emphasis, with the aim of increasing the high-frequency component of the input signal, which contains more information. While increasing the energy of the high-frequency part of the input signal, the pre-emphasis can keep the energy of the low-frequency part of the input signal unchanged. After that, unified framing and windowing are performed on the data. The window function is determined using Equation (1).

(1) $$\{\begin{array}{c} Wn,\alpha =\left(1-\alpha \right)-\alpha *\cos\left[\frac{2\pi n}{N-1}\right],0\leq n\leq N-1\\ 0,n=else \end{array}$$

where *N* is the length of the Hamming window, *α* = 0.46 and n is the sampling point.
**Algorithm 1** Loop padding(1)Input: *y_n_*: raw audio;(2)Output: *Y_new_*: after the loop padding of the raw audio;(3)*n* ← number of original audios samples;(4)*n_all_* ← specified number of audios samples = 4 × 16,000;(5)*cnt* ← 0;(6)if *n* > *n_all_* + 1 then(7)return *Y_new_* [: *n_all_*](8)else(9)*Y_new_* ← np. zeros (*n_all_*)(10)*Y_new_* [: *n*] ← *y_n_* [: *n*](11)For i in range (*n*, *n_all_*) do(12)*Y_new_* [i] ←*y_n_* [*cnt*](13)*cnt* ←*cnt* + 1(14)if *cnt* == *n* then(15)*cnt* ← 0(16)return *Y_new_*(17)end if

### 3.2. Feature Extraction

For feature extraction of audio signal processing [26], the first step is to analyze and extract the feature parameters that can represent the essence of the signal. Only with feature parameters can these feature parameters be used for effective processing. According to the different methods of extracting parameters, audio signal analysis can be divided into time domain, frequency domain, inverse frequency domain, and other domain analysis methods. The analysis method used in this article is frequency domain analysis, and a detailed feature extraction process is introduced in the following text.

Log-spectrogram feature extraction: The spectrum of each frame is obtained with a fast Fourier transform (FFT) method, and then the complete spectrum is obtained by superposition along the specified dimension. The interval between every two frames is generally 20–70% of the frame length. By increasing the length of a frame, the information in the frequency domain becomes more accurate. The time-series information of signal changes cannot be obtained by using fast Fourier transform directly on non-stationary signals. For example, over a period, many signals first appear and then disappear. Therefore, it is impossible to use the fast Fourier transform directly to judge the order of different signals. The FFT reflects the change in the signal over time by taking the signal a little bit at a time and applying the discrete Fourier transform method. Finally, take the logarithm to extract the log-spectrogram characteristics of the signal, and use the spectrum as the input to the network model. The length of the FFT window of the logarithmic spectrogram is 400, and the frame shift bits are 160. The final extracted log-spectrogram matrix size is 201 * 401.

Log-Mel spectrogram feature extraction: Extract log-Mel spectrogram features from the original signal data because the network uses the log-Mel spectrogram as the input to the model rather than the basic signal. Use Equation (2) to determine the Mel-scale frequency and convert the spectrogram of each frame stack obtained above to the spectrogram under the Mel scale through Equation (2).

Finally, zero mean normalization is performed as shown in Equation (3) to accelerate the convergence of weight parameters at each layer of the network during the training backpropagation process. The FFT window length of the log-Mel spectrogram is 400, the frame offset is 160, and the number of Mel filter banks is 80. The final extracted log-Mel matrix size is 80 * 401.

(2) $$\mathrm{Mel}\left(f\right)=2595*\mathrm{lg}\left(1+\frac{f}{700}\right)\;$$

(3) $$X=\frac{X_{i}-\mu }{\sigma }$$

where f is the original signal sampling frequency, *μ* is the mean value of the original data, *σ* is the standard deviation of the original data and *X* is a standardized feature.

### 3.3. Dual-Branch Residual Network

**Figure 3:**
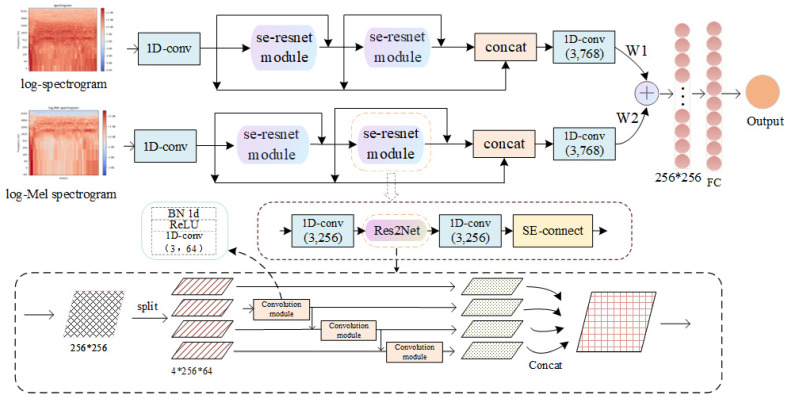
The detailed network architecture of the dual-branch residual network.

The dual-resnet structure (Figure 3) input data proposed in this paper are log-spectrogram and log-Mel spectrogram. Since multiple features have better frequency domain information representation compared to a single feature, we absolutely use a dual-branch network to extract two features separately, rather than using the method of concatenating two features and inputting them into a single-branch network. And we consider adding Res2Net module in the dual-branch network, which can not only obtain a different receptive field, but also improve the effective receptive field, thus avoiding the repeated use of redundant information, and can effectively extract frame-level features and segment-level features.

The first layer 1D convolution kernel size is set to 2 * 401 to correspond to the sound signal’s log-Mel spectrogram and log-spectrogram dimensions. The second and third layers are se-resnet modules. They use skipping connections to ensure the reusability of features, then concatenate the outputs of the first, second, and third layers. Finally, the model uses the self-learned W1 and W2 weights to “add” the two branches (W1 and W2 constantly modify their values during the learning process to achieve optimization) so that the features of the two branches are fused to the “softmax” classifier to output the classification result. The softmax function is used for multiple classification tasks. Literally, the outputs of multiple neurons are mapped to the (0, 1) interval through the softmax function, and the sum of these output values is 1. After obtaining the probability of each element through the softmax function, we output the index of the maximum value among all elements to obtain the final category. We need to use the argmax function to obtain the position of the maximum probability.

The se-resnet module consists of two layers of 1D convolutional layers (convolution kernel size is 3 * 256, step size 1), a layer of Res2Net, and a self-attention layer. In Res2Net, the input is first divided into tensor blocks (specific number = 4) along the specified dimension (dim = 1), and a set of convolution modules that first extract elements from a set of tensor blocks. Then, the output characteristic map of the previous tensor block is sent to the next set of convolution modules along with another set of input tensor blocks. This process is repeated several times until all input feature maps have been processed. Each tensor block is connected using a similar residual method after passing through the convolution module. Finally, the four tensor blocks are connected to fully integrate information, and the output obtains a larger receptive field. Obviously, the input of the fourth set of convolution operations includes the first two sets of input features. So Res2Net can extract features of a different receptive field and multiple scales, and can effectively extract global features and local features. The acquisition of feature information is different under a different receptive field. A small receptive field may see more detail information. For example, in audio features, a small receptive field focuses more on frame-level features, while a large receptive field can acquire overall structural features to facilitate the network to locate the start and end positions of sound. In the self-attention layer [27], the specified dimension of the input (dim = 2) is first averaged. Then, the features are compressed and stretched through two fully connected layers to obtain the self-attention vector and compared with the input element-by-element multiplied to obtain the attention-weighted feature vector.

### 3.4. Training the Network

The customDataset() function and DataLoader() function are used to obtain audio data from the training and validation sets. The obtained data are input into the dual-branch residual network model built above to start training. The training data are processed through the network to obtain the final output. The softmax function is used to normalize the ten values in the output array, and then the argmax function is used to return the index value of the maximum value in the output array, which is the category name. If the output category name is the same as the label, it indicates accurate classification. The number of accurate classifications is added by one, and the final accuracy rate is number of accurate classifications/number of training samples. Training logs are printed every 10 batches, and after one round of training, validation is performed and the model is preserved. During validation, the output of each batch is counted, and finally the average value is calculated.

The dual-branch residual network is trained with the cross-entropy using adaptive moment estimation (Adam). Adam is essentially RMSprop with momentum term, which dynamically adjusts the learning rate of each parameter by using the first and second moment estimates of the gradient. Its main advantage is that after offset correction, the learning rate of each iteration has a certain range, making the parameters more stable. The trainable parameter of our model is 2.4 M, and we set the initial value of learning rate to 0.001 and the training epochs to 120. In addition, we apply batch normalization to all convolutional layers, playing a function similar to dropout to accelerate training speed.

## 4. Experiment and Result

### 4.1. Datasets

**Figure 4:**
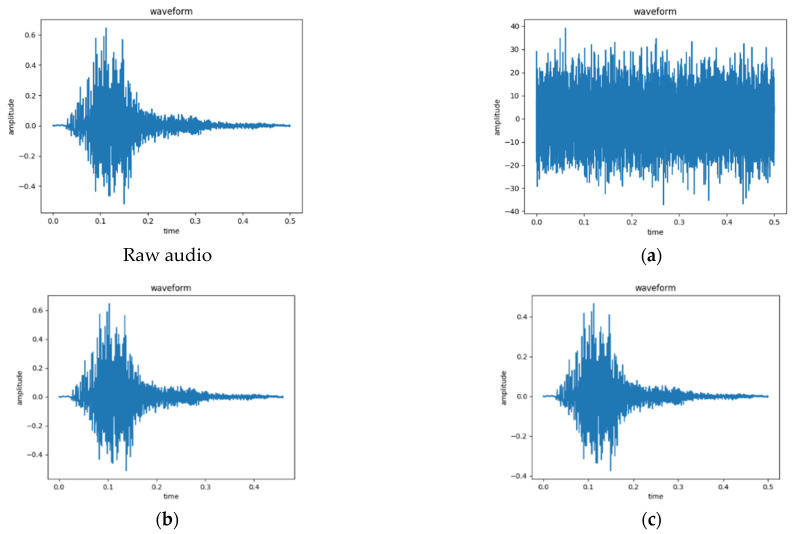
Data enhancement. (**a**) add Gaussian noise; (**b**) time-stretching; (**c**) volume changes.

Currently, the most used datasets for ESC include UrbanSound8K, ESC-50 [28], and ESC-10. This paper uses the public dataset UrbanSound8K for automatic urban environmental sound classification research. This dataset is divided into ten sound categories: air conditioner, car horn, children playing, dog bark, drilling, engine idling, gunshot, jackhammer, siren, and street music. There are many ways to prevent model overfitting, such as through data enhancement [29], early stop training, dropout regularization [30], and so on. This paper adopts the method of data enhancement in time domain. Gaussian white noise, time-stretch and volume-change methods are used to enhance the data of the training set. The audio clips from the UrbanSound8K dataset are randomly selected, and the original audio is called X; Figure 4a shows the enhanced sound segment waveform of the noise. By specifying a minimum signal-to-noise ratio of 10 and a maximum signal-to-noise ratio of 50, the data enhancement probability is 0.6, and the signal-to-noise ratio is randomly selected from 10 to 50 for each enhancement. The random signal-to-noise ratio is multiplied with a randomly generated Gaussian distribution with a standard deviation of 1 to obtain random Gaussian white noise, and then the original audio X with Gaussian white noise is added to obtain the enhanced data.

Figure 4b shows the enhanced sound segment waveform through the time-stretching method. The audio speed is changed through linear interpolation. First, the new audio length L is obtained by dividing the original audio length by the new sampling rate (the new sampling rate values are 0.9~1.1). Returning L evenly spaced arrays between the interval 0 and the original audio length is called X_b_; regenerating them into a starting point value of 0, an ending point value of the original audio length, and an equal difference array with a step size of 1 is called X_c_. Finally, the enhanced audio is obtained by fitting the audio curve with one-dimensional linear interpolation (X_b_, X_c_, X).

Figure 4c shows the waveform of the sound segment enhanced by volume changes. The volume enhancement is obtained by multiplying the original audio with random gain to obtain the changed data, as shown in Equation (4).

(4) $$Y=X*10^{G/20}$$

where *X* is the original data, *G* is random gain, with a value range of −15~15 and *Y* is the data after random gain.

### 4.2. Comparison between Loop Padding and Zero Padding

**Figure 5:**
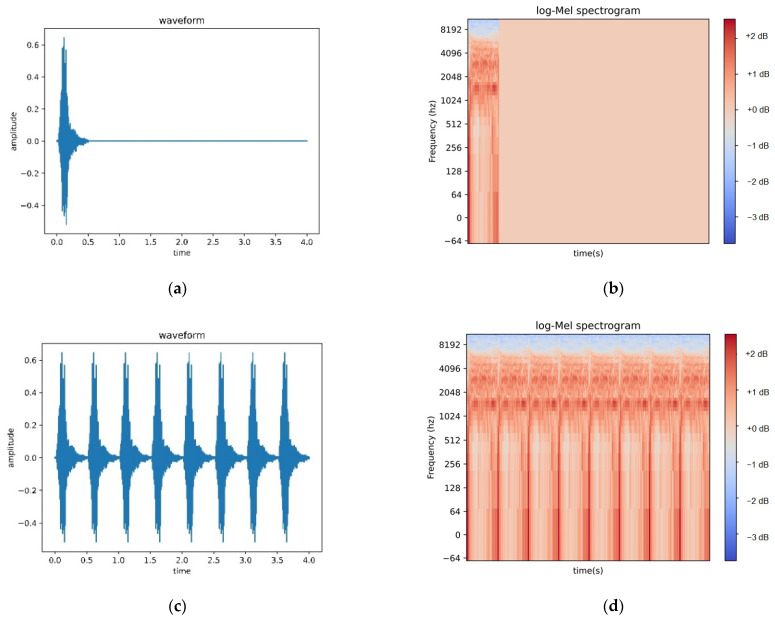
(**a**) Zero-padding waveform; (**b**) zero-padding log-Mel spectrogram; (**c**) loop-padding waveform; (**d**) loop-padding log-Mel spectrogram.

To verify the usability of the loop-padding method proposed in this paper, we compare it with the most commonly used zero-padding method. In this experiment, we used 8732 audio dates from UrbanSound8K as the experimental dataset. The training data are 6112, the validation data are 1746, and the test data are 874, with a ratio of nearly 7:2:1 for the training, validation, and testing sets. The accuracy of this paper is based on the accuracy of the test dataset. Figure 5 is a comparison diagram between zero-padding and loop-padding methods:

(a) Is the time-domain waveform of the original data after zero padding, and the NumPy. Pad ( ) function is used to unify the original signal to 4 s by zero padding.
(b) Is the log-Mel spectrogram after the zero-padding method, and the log-Mel spectrogram feature extraction mentioned above was carried out on Figure 5a.
(c) Is the time-domain waveform of the original data after loop padding, loop padding the original signal through Algorithm 1 proposed above and unifying it into 4 s.
(d) Is the log-Mel spectrogram after the loop-padding method, and the log-Mel spectrogram feature extraction mentioned above was carried out on Figure 5c.

Table 1 shows the comparison results. Compared with the zero-padding method, the classification accuracy of the loop-padding method proposed in this paper has been improved to a certain extent. We conducted experiments using different network models and found that loop padding, whether combined with the network model proposed in this article, or with the simplest convolutional neural network or dilated convolutional neural network, has a high improvement in classification accuracy compared to the zero-padding method, and the combination of the loop-padding method and the network model proposed in this article has the greatest improvement in classification accuracy. The reason is that when dealing with one-dimensional-dynamic audio signals, the zero-padding method fills the original audio data to a certain length while ignoring the time characteristics of the audio signal. When the log-Mel spectrogram after the zero-padding method is input into the subsequent network feature extraction, it either contains more useless information or the amount of useful information remains unchanged, and even some harmful information is mixed to reduce classification accuracy. However, after the loop-padding method, the log-Mel spectrogram provides more useful information for subsequent feature extraction while ensuring the temporal characteristics of the audio signal.

### 4.3. Proposed Model

To verify the effectiveness of the feature fusion of the dual-branch network, the log-Mel spectrogram and log-spectrogram were respectively input into the single-branch residual network for experiments. To ensure the accuracy of the ablation experiment, the single-branch residual network structure, and the double-branch—arbitrary branches of the residual network remain consistent. Feature concatenate–single branch uses the early fusion method to input the fused features into the single-branch network for training. Feature fusion–double branch is the network structure proposed in this paper. The results are shown in Table 2. It can be seen that the feature fusion accuracy of the dual-branch network is higher than that of the feature concatenate–single branch. Due to early fusion, its feature resolution is higher and contains more detailed information, but due to less convolution, its semantics are lower and there is more noise. It can also be seen that feature fusion has higher accuracy than single-feature classification. Environmental sound has the characteristics of nonlinearity and multiple sound sources, so a single feature cannot fully characterize the audio signal. In addition, the log-spectrogram can intuitively reflect the changing characteristics of the signal over time at different frequencies. It also combines the log-Mel spectrogram to compensate for the sensitivity of the spectrum to low-frequency feature changes and the sluggishness of high-frequency feature changes, making the audio features represented more comprehensive and accurate.

**Figure 6:**
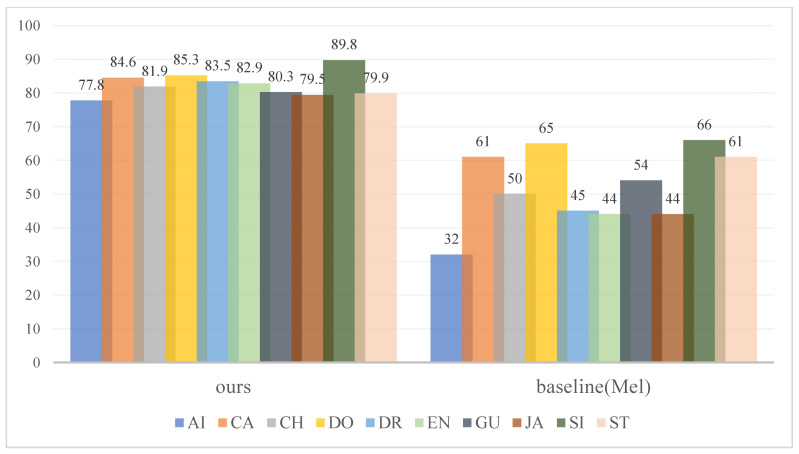
Classifier accuracy (%) in UrbanSound8K database for different sound categories: Air conditioner (AI), Car horn (CA), Children playing (CH), Dog bark (DO), Drilling (DR), Engine idling (EN), Gunshot (GU), Jack hammer (JA), Siren (SI) and Street music (ST).

Based on the UrbanSound8k dataset, Figure 6 reports the performance of the Mel baseline and the proposed approach. In addition, from the perspective of classification accuracy, the network proposed in this paper has different degrees of improvement compared to the Mel baseline system in all ten categories. In particular, for “Air conditioner”, “Children playing”, “Drilling”, “Engine idling” and “Jack hammer”, the classification accuracy of these five categories has significantly improved compared to the Mel baseline system. The reason is that Mel features pay more attention to the low-frequency segmentation of audio and are more sensitive to low-frequency feature changes, while ignoring high-frequency features in “Drilling”, “Engine idling” and “Jack hammer”, resulting in low classification accuracy. However, this article combines log-spectrogram to make up for the disadvantage of the Mel spectrogram being insensitive to changes in high-frequency features; this makes the representation of audio features more comprehensive and accurate, thereby improving classification accuracy.

The system model proposed in this paper is used for sound classification and compared with other state-of-the-art environmental sound classification methods. Experiments were conducted on two datasets, UrbanSound8k and ESC-50. By comparing the results of CNN and the results of the dilated CNN in Table 3, it can be seen that the introduction of the dilated CNN into the ESC problem has achieved better results, which proves that increasing the receptive field is effective. And we compared the time required for these models to infer 874 test data. Although we require longer model inference time compared to some models, our model achieves higher classification accuracy, further proving that our proposed model can be effectively applied to environmental sound classification tasks and can achieve ideal classification results.

## 5. Conclusions

According to the sound signal, the urban environmental sound classification system is expected to become an essential tool for constructing a “smart city”, and it has crucial use value for the management of the city and the construction of security. This paper proposes a dual-branch network structure, which not only increases the receptive field and obtains multi-scale features, but also better integrates the key feature information in the log-Mel spectrogram and log-spectrogram to solve the problem that a single-feature extraction method cannot accurately and completely capture audio features. In addition, a loop-padding method is proposed in the data processing section to obtain a broader range of audio temporal feature information more accurately. At the same time, in order to prevent overfitting in the process of model training, this paper uses the time-domain data enhancement method to expand the number of datasets by randomly adding Gaussian white noise, time stretch, and volume change. Finally, according to the experimental results, based on the UrbanSound8K dataset, the accuracy reached 82.6%, and based on the ESC-50 dataset, the accuracy reached 86.3%, confirming the advantages of our method.

In our future work, more machine learning methods, such as semi-supervised learning [34,35], will be explored to reduce the labelling cost of massive datasets in the ESC domain.

## Figures and Tables

**Table 1:** Comparison between loop padding and zero padding.

Padding Methods	Model	Sample Data Proportion	Accuracy (%)
zero padding	Dual-resnet	7:2:1	75.8%
loop padding	Dual-resnet	7:2:1	82.6%
zero padding	CNN	7:2:1	73.9%
loop padding	CNN	7:2:1	76.1%
zero padding	Dilated convolutions	7:2:1	77.4%
loop padding	Dilated convolutions	7:2:1	79.6%
zero padding	EnvNet	7:2:1	78.2%
loop padding	EnvNet	7:2:1	80.3%

**Table 2:** Experimental result.

Method	Single Branch	Double Branch
Log-Mel spectrogram	74.9%	-
Log-spectrogram	71.4%	-
Feature concatenate	76.8%	-
Feature fusion	-	82.6%

**Table 3:** Comparison with other methods on the UrbanSound8k, ESC-50 dataset.

Model	Representation	UrbanSound8k		ESC-50
		Accuracy (%)	Inference Time (s)	Accuracy (%)
Baseline system [25]	Mel-Frequency Cepstral Coefficients (MFCC)	69.1	-	-
CNN [9]	Mel spectrogram	73.2	129.73	65.1
Dilated CNN [31]	Mel spectrogram	78.3	127.31	83.9
EnvNet [32]	Original waveform	77.6	118.56	83.5
CRNN [33]	Original waveform	79.0	115.60	-
Ours	Log-Mel spectrogram Log-spectrogram	82.6	123.39	86.3

## Data Availability

The dataset employed in this study is available upon request at the link https://zenodo.org/record/1203745/files/UrbanSound8K.tar.gz (accessed on 13 June 2023).
